# Cardiotoxicity: Myocardium or Endothelium

**DOI:** 10.7759/cureus.2994

**Published:** 2018-07-18

**Authors:** Raúl A Montañez-Valverde, David Hurtado-de-Mendoza, Arturo Loaiza-Bonilla

**Affiliations:** 1 Cardiovascular Division, Beth Israel Deaconess Medical Center/Harvard School of Medicine, Boston, USA; 2 University of Miami Miller School of Medicine, University of Miami Miller School of Medicine/Jackson Memorial Hospital, Miami, USA; 3 Medicine, Hematology and Oncology, Cancer Treatment Centers of America, Philadelphia, USA

**Keywords:** cardio-oncology, oncology, medical oncology, adverse effect, drug adverse effects

## Abstract

Endothelial biomarkers are gaining interest in the stratification of cardiovascular risk and early diagnosis of cardiotoxicity secondary to antineoplastic drugs. Interestingly, some drugs, such as anthracyclines, have been recently associated with vascular damage, which reveals the pivotal role of research in identifying biomarkers that could potentially be included into more specific cardiotoxicity risk scores. An extensive report of the incidences of cardiovascular adverse effects of oncologic drugs is presented, with the main purpose of highlighting not only the risk of developing heart failure but also the importance of associated vascular adverse effects (i.e., hypertension, venous, and arterial thrombosis) experienced by patients in the post-chemotherapy phase.

## Editorial

Due to the ongoing research on a clinically relevant subject such as the cardiotoxic effects of oncologic drugs as exemplified by Finkelman et al. in the article “Arginine-Nitric Oxide Metabolites and Cardiac Dysfunction in Patients With Breast Cancer” [1], we wanted to point out some of the challenges faced when trying to stratify such cardiovascular risk. An extensive report of the incidences of cardiovascular adverse effects of oncologic drugs is presented, with the main purpose of highlighting not only the risk of developing heart failure but also the importance of the associated vascular adverse effects (i.e., hypertension, venous and arterial thrombosis) experienced by patients in the post-chemotherapy phase.

The most confusing aspect of the definition of cardiotoxicity is whether only clinical, clinical and subclinical, or only subclinical events should be considered [2]. Another major clinical difficulty is related to the timing of detection of myocardial damage, since a measurement of left ventricular ejection fraction at some time point during or after therapy is clearly insufficient [3]. If a biomarker allowed us to better stratify patients, and to initiate cardioprotective therapy in those patients at the earliest sign of toxicity, new efficient interventions could substantially improve outcomes [3].

Endothelial biomarkers are gaining interest; it is known that bevacizumab decreases endothelial nitric oxide synthase activity, which may stimulate plasminogen activator inhibitor-1 expression, leading to an increased risk of hypertension [4]. Finkelman et al. reported that plasma levels of endothelium biomarkers such as arginine, citrulline, and dimethylarginine were altered during the early phase of treatment with anthracycline-based chemotherapy [1]. As mentioned by Lenihan [3], if the predominant mechanism of early cardiotoxicity related to anthracycline administration were actually vascular in nature, it would substantially change potential prevention and treatment strategies. The severity of these adverse effects may not present in an early catastrophic manner as it would likely happen secondary to direct cardiotoxicity (e.g., systolic dysfunction), but they could highly impact the long-term prognosis.

Herrmann J et al. proposed a Cardiotoxicity Risk Score (CRS) that included a medication-related risk depending on incidence rates of cardiotoxicity [5]. The cut-off values for these categories were >15% (high), 15% – 5% (moderate), <5% (mild) and <1% (rare), which we used to categorize the risk in this brief communication. A thorough review of the literature in Dailymed (US National Library of Medicine), UpToDate®️, US Food and Drug Administration (FDA) approved drug online registry and Pubmed (Medical Subject Headings terms: "Adverse effects", "Drug-related Side Effects and Adverse Reactions") was conducted, looking for incidences of cardiotoxicity of oncologic drugs. It is pertinent to mention that the heterogeneity of the information made this analysis more challenging. For e.g., the time point when cardiotoxicity becomes clinically evident varies substantially; this is the reason why different follow-up periods have been used. Furthermore, the different types of populations (e.g., naïve vs. relapse), different regimens, patient’s Eastern Cooperative Oncology Group (ECOG) performance status, and disease stage and grade, add complexity to the conclusions achieved (Table 1). Literature is vast regarding the data here cited. Due to the nature of this brief editorial, only the most relevant references have been listed; however, the authors would be more than pleased to e-mail the full list of sources to the readers who do require so.

**Table 1:** Reported incidences (%) of cardiovascular adverse effects of oncologic drugs

Drug	HF	Prolonged QTc	A-fib	MI	HTN (all grades)	VTE	Arterial thrombosis
Anthracyclines (Doxorubicin)		14.0					
Doxorubicin 400 mg/m2	5.0						
Doxorubicin 550 mg/m2	26.0						
Doxorubicin 700 mg/m2	48.0						
Trastuzumab	20.1^(a)^				4.0		
Ifosfamide			1.0				
Ifosfamide <10 g/m2	0.5						
Ifosfamide 12.5–16 g/m2	17.0						
Cyclophosphamide	28.0						
Clofarabine	27.0				13.0		
Carfilzomib	25.0				42.0	2.0	
Sunitinib	18.0	4.0			27.0	3.0	1.4
Pazopanib	11.0	2.0		2.0	40.0	5.0	0.3
Sorafenib	8.0			3.0	15.3		1.7
Trametinib	11.0				15.0		
Dabrafenib	9.0	2.0			4.0		
Docetaxel	13.0		1.0	1.7			
Pertuzumab	7.0						
Daunorubicin	10.1						
Bevacizumab	4.0^(b)^			1.5	23.6	33.0	6.0
Ramucirumab					16.0		2.0
Imatinib mesylate	2.7		1.0	0.1	4.0		
Dasatinib	4.0	3.0	1.0	4.0	1.0		
Lapatinib	1.5	16.0					
Nilotinib	1.0	10.0		9.0	11.0		15.0
Bortezomib	5.0		1.0				
Cisplatin						8.5	2.0
Osimertinib	1.9	2.9					
Ado-trastuzumab emtansine	2.0				5.0		
Lenvatinib	2.0				73.0		5.0
Cabozantinib					61.0	9.0	2.0
Ponatinib	9.0		7.0	35.0	74.0	6.0	
Lenalidomide	1.0			1.7	8.0	10.0	
Arsenic trioxide		40.0			10.0		
Rituximab					12.0		
Temsirolimus	1.0				7.0	2.0	
Everolimus	1.0		1.0		30.0		
Sirolimus					49.0		
Ibrutinib			9.0		17.0		
Alemtuzumab					14.0		
Gefitinib					9.0		1.0
Ibritumumab tiuxetan					7.0		
Daratumumab					10.0		
Pembrolizumab					7.0		
Ofatumumab					5.0		
Paclitaxel	1.0			5.0		1.0	
Thalidomide				3.0		22.0	
Erlotinib				2.3		11.0	
Vorinostat		6.0				8.0	
Atezolizumab						10.0	
Panitumumab						3.0	
Nivolumab						3.0	
Cetuximab				2.0			
Cytarabine [Liposomal]					6.0		
5-fluorouracil						6.0	
Capecitabine			5.0			8.0	
The incidences are the highest reported in literature.							
(a) When used in combination with anthracyclines and cyclophosphamide. (b) In patients receiving concurrent anthracyclines.							
A-fib: atrial fibrillation; HF: heart failure; HTN: hypertension; MI: myocardial ischemia; QTc: corrected QT interval; VTE: venous thromboembolism.							

**Figure 1:**
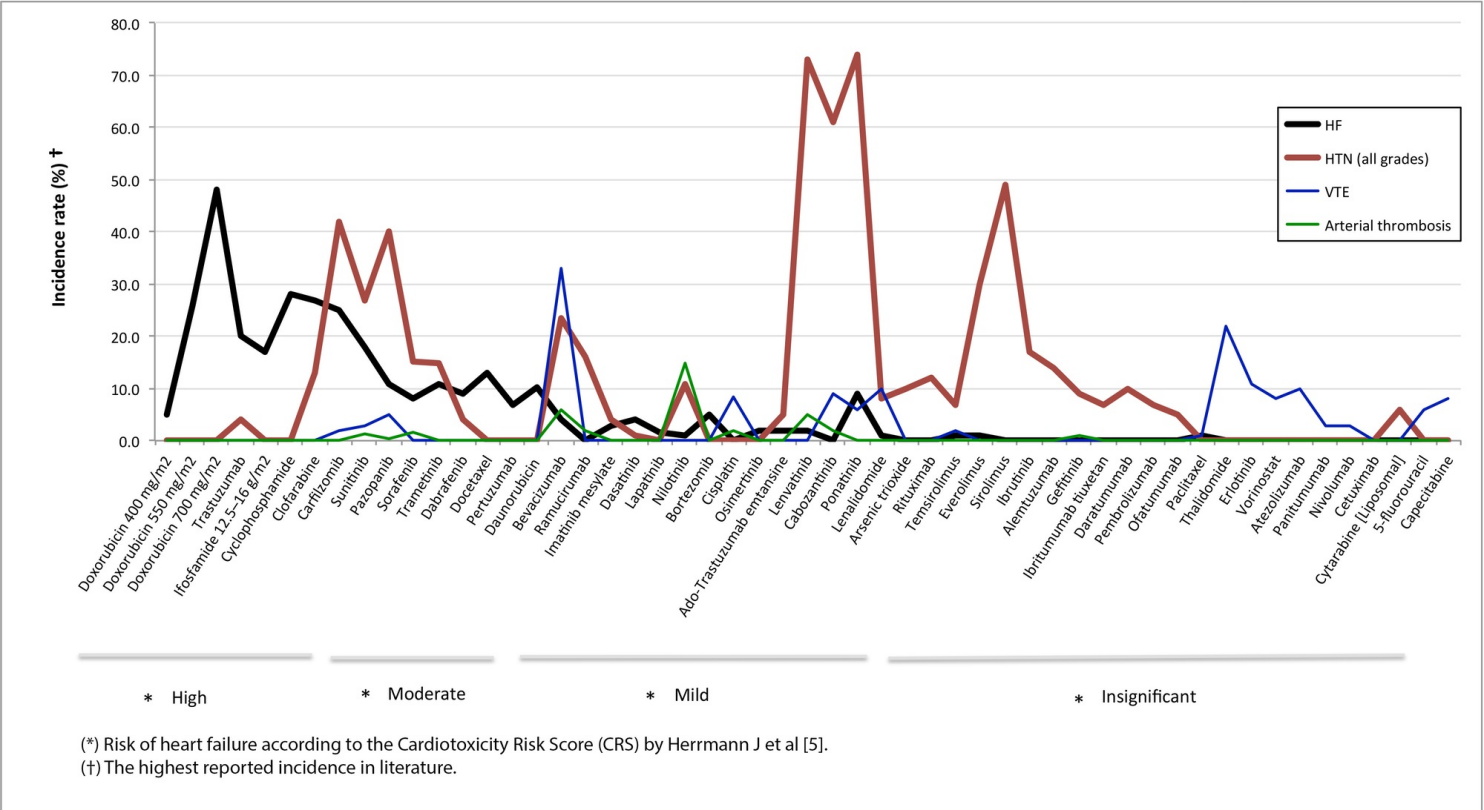
Cardiovascular adverse effects of oncologic drugs: myocardium or endothelium HF: heart failure; HTN: hypertension; VTE: venous thromboembolism.

As depicted in Figure 1, some drugs primarily affect cardiac function (i.e., anthracyclines and trastuzumab), vascular function (e.g., 5-fluorouracil and capecitabine), or both (e.g., bevacizumab and sunitinib); information concordant with current accepted and potential mechanisms [5]. Tyrosine kinase inhibitors (TKIs) present a risk of vascular damage associated with moderate (i.e., sunitinib, pazopanib, sorafenib, trametinig) or mild (i.e., levantinib, carbozantinib, ponatinib) risk of myocardium injury. Likewise, monoclonal antibodies (Ab) such as bevacizumab (VEGF Ab) and ado-trastuzumab (HER2 Ab) have a mixed effect, whereas rituximab (CD20 Ab), alemtuzumab (CD52 Ab) and daratumumab (CD38 Ab) report almost exclusive vascular adverse effects. Interestingly, some drugs, such as anthracyclines [1], have been recently associated with vascular damage, which reveals the importance of research in identifying new biomarkers that could be used to improve cardiotoxicity risk scores.

Through this manuscript, we would like to reinforce the idea that when addressing cardiotoxicity, not only should the myocardium be considered in our evaluation but also the endothelium. The latter could play an important role in how our patients face the early and late post-chemotherapy phase, thus helping to stratify patients' risks and optimize their management.
